# Femtosecond Laser Assisted Deep Anterior Lamellar Keratoplasty Outcomes and Healing Patterns Compared to Manual Technique

**DOI:** 10.1155/2015/397891

**Published:** 2015-10-18

**Authors:** Jorge L. Alio, Ahmed A. Abdelghany, Rafael Barraquer, Laila M. Hammouda, Ahmed M. Sabry

**Affiliations:** ^1^Vissum Corporation, 03016 Alicante, Spain; ^2^Division of Ophthalmology, Miguel Hernandez University, Alicante, Spain; ^3^Ophthalmology Department, Faculty of Medicine, Minia University, Minia 61111, Egypt; ^4^Barraquer University Institute, Autonomous University of Barcelona, Barcelona, Spain

## Abstract

The purpose of the study is to report the visual, refractive, and wound healing pattern outcomes of femtosecond assisted deep anterior lamellar keratoplasty (DALK) compared to the conventional manual technique. DALK was performed on 50 eyes of 47 advanced keratoconus patients. The patients were divided into two groups, 25 eyes each, depending on whether femtosecond assisted or manual DALK technique was performed for the side cut of the procedure only. Patients were followed up at 1 month, 6 months, and 1 year for visual acuity, clinical refraction, corneal cylinder, date of suture removal, and side cut corneal healing pattern according to new grading classification of the side cut scar (Grade 0 = transparent scar, 1 = faint healing opacity, 2 = evident healing opacity, 3 = significant opacity with some cosmetic imbalance, and 4 = highly significant opacity with very significant cosmetic imbalance). Outcomes are reported at one year. In conclusion, femtosecond assisted and manual DALK show comparable visual and refractive outcomes but femtosecond assisted DALK shows more evident corneal wound healing patterns at the side cut. This observation may indicate that an activated cornea wound healing might allow earlier suture removal when femtosecond technology is used to perform the side cut for DALK.

## 1. Introduction

Deep anterior lamellar keratoplasty (DALK) is a surgical procedure in which a diseased corneal stroma is excised until the Descemet membrane (DM) or as close as possible followed by transplantation of the donor corneal button free from DM and endothelium. This procedure can be considered the first line surgical choice for corneal stromal diseases with intact endothelium [1], better than penetrating keratoplasty (PK) as far as DALK is associated with a lower risk of graft rejection, secondary glaucoma, complicated cataract, and postoperative long term loss of endothelial cells [2].

A report from the American Academy of Ophthalmology concluded that DALK was equivalent to PK in terms of graft survival, best corrected visual acuity, and refractive errors, but DALK may be the superior procedure regarding the preservation of corneal endothelial cell density [3]. Femtosecond assisted DALK has been suggested as a more advanced and probably better procedure for the performance of DALK surgery, in particular the side cut [4].

Corneal grafting techniques are affected by many variables, biological, immunological, biomechanical, surgical, and technological variables, which make this procedure intrinsically variable. This variability of outcomes affects not only the visual and refractive outcomes but also the biological performance of the tissues affected by the graft and by the surgical trauma and, overall, the visual recovery of the patient [5].

The femtosecond laser (FSL) is able to make precise corneal incisions with customized graft edges and lamellar planes for both donor and recipient corneas [6]. The use of femtosecond laser due to its precision and control in sizing of the donor and recipient corneal buttons might help in the control of many of the previously mentioned variables making corneal grafting surgery a better and more controllable technique with better outcomes [5].

In addition, the different patterns that can be performed with the FSL allow an excellent apposition of the tissue that result in rapid wound healing, which may lead to earlier removal of the suture and faster patient recovery [7].

The aim of our study is to compare manual and femtosecond assisted DALK (Fs-DALK) in terms of refractive and visual outcomes and to ascertain whether corneal wound healing patterns appear in Fs-DALK different to those that are observed in the manual technique.

## 2. Materials and Methods

### 2.1. Study Design

Prospective and retrospective consecutive comparative clinical series of cases. The study was carried out in accordance with the Declaration of Helsinki [8] and was approved by the Ethical Committee (CEIC) of our institution in Alicante.

### 2.2. Inclusion Criteria

Patients included in this study underwent DALK due to advanced keratoconus. All patients were free of any other ocular comorbidity other than the corneal ectatic disorder leading to the indication of corneal graft. Patients were matched for age and sex to create equivalent groups for the purpose of the study. If complications such as perforation during the stromal dissection happened, the case was excluded from the investigation and replaced by another with similar profile.

We divided the patients into two groups according to the technique used to perform the side cut in the donor and the recipient cornea.

#### 2.2.1. Group 1 Femtosecond Assisted DALK

25 eyes of 22 patients underwent femtosecond laser mushroom configuration DALK between January 2010 and May 2013. All surgeries were performed by the same expert surgeon (JLA) at Vissum Instituto Oftalmologico, Alicante, Spain.

#### 2.2.2. Group 2 Manual DALK

10 eyes of 10 patients underwent manual trephine straight-edge configuration DALK between May 2012 and January 2013. Other 15 cases of manual DALK were performed by another expert surgeon (RB) during the same period of time at Institut Universitari Barraquer, Universitat Autónoma de Barcelona, Spain. These cases were analyzed retrospectively at one year of the follow-up using the same observational protocol as in the other cases. Both surgeons followed the same surgical and postoperative protocol.

### 2.3. Surgical Technique

Manual trephine straight-edge configuration DALK was performed using the Melles technique implemented by the injection of air in the residual stroma left by the manual dissection to better accomplish the dissection of the deep stroma. The dissection was performed in all cases down to the Descemet layer or leaving minimal amounts of residual stroma tissue in case that big bubble was not accomplished. The donor cornea was in all cases the same diameter as the recipient button (8 mm of diameter). The donor was secured to the recipient with a double torque-antitorque 16 bites' continuous suture.

Femtosecond laser mushroom configuration DALK was performed by a 60 KHz Intralase Femtosecond Laser (IntraLase, Abbott Medical Optics, Santa Ana, California, USA). Only the side cut was performed. The corneal stroma was excised and completed down to the Descemet membrane or to deepest stromal layers assisted by the injection of air (big bubble technique).

**Figure 1:**
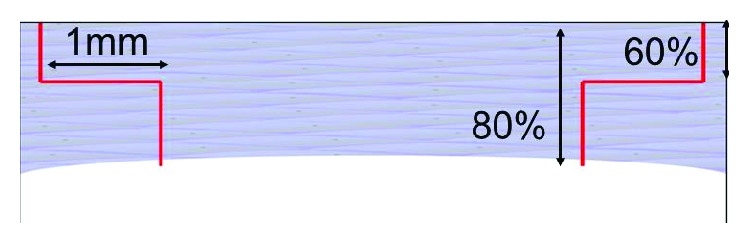
Mushroom configuration.

For the side cut a full-thickness mushroom configuration cut was made on the donor cornea first and then a nonpenetrating mushroom configuration on the recipient, using the FS laser system. The energy used was 2 to 2.3 mJ depending on the case. In the recipient cornea, the depth of the anterior side cut was about 60% of the thinnest corneal pachymetry, and the depth of the posterior side cut was about 80% of the thinnest corneal pachymetry, leaving a ring lamellar cut of 1 mm (Figure 1).

In the donor cornea, the Descemet membrane (DM) and endothelium were debrided in all cases of both groups assisted by trypan blue dye (vision blue dye).

### 2.4. Postoperative Management

- Topical antibiotic eye drops: cetraflux 3 mg/mL (ciprofloxacin) 4 times daily for 1 week till complete epithelial healing and removal of contact lens (once daily after 1 week if epithelium did not completely heal or contact lens were still not removed).
- Topical steroid eye drops: Pred Forte (prednisolone acetate) 8 times daily for 1 week and then tapering gradually through 1 month and finally once daily forever.
- Contact lens for 1 week for comfort of the patient and till complete epithelial healing.
- Cycloplegic eye drops twice daily for 3 days.
- Tear substitute if needed.

Postoperatively all patients were followed up by the surgeon and a cornea specialist at the cornea units of each institution at 1 month, 6 months, and 1 year for visual acuity (uncorrected and best corrected) and corneal cylinder studied by corneal topography map. Suture removal was not performed in any case before the end of the 12th month of follow-up. The side cut corneal healing pattern was evaluated according to a grading system established for the purpose of this investigation. The grading was performed as observed and registered photographically by slit lamp photography with illumination at 45° light angle of incidence concerning the slit lamp observation optics placed orthogonal to the corneal vertex as observed by the first Purkinje reflex. The grading of the scar was performed as follows: Grade 0 = transparent scar, Grade 1 = faint healing opacity, Grade 2 = evident healing opacity, Grade 3 = significant opacity with some cosmetic imbalance, and Grade 4 = highly significant opacity with very significant cosmetic imbalance. The same investigator (AA) performed all the slit lamp side cut wound healing gradings of this investigation.

### 2.5. Statistical Analysis

SPSS V.21 was used for the analysis. The postoperative outcomes between manual and femtosecond groups were compared using Mann-Whitney *U* test. For all the analysis, *P* value < 0.05 was considered statistically significant.

## 3. Results

### 3.1. Baseline Characteristics

There were no significant differences in age or gender between the manual and femtosecond groups (*P* = 0.211). A big bubble was achieved following the stromal dissection in 20 of the cases of the Fs-DALK and in 21 of the manual cases.

### 3.2. Visual Outcomes

There were no significant differences in uncorrected distant visual acuity (UCDVA) and best corrected distant visual acuity (BCDVA) at 1 month, 6 months, and 1 year between the two groups (Table 1).

### 3.3. Corneal Topography Cylinder Analysis

There were no significant differences in corneal cylinder taken by corneal topography at 1 month, 6 months, and 1 year between the two groups (Table 2).

### 3.4. Healing Pattern at the Side Cut between the Donor and Recipient Cornea

**Figure 2:**
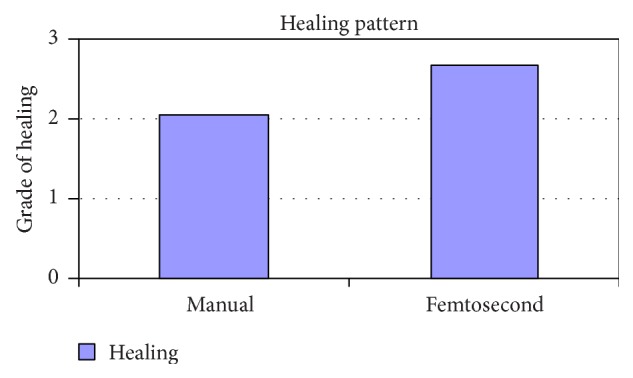
Healing in manual and femtosecond DALK. Healing is more evident in femtosecond assisted DALK.

Slit lamp pictures of all cases were reviewed by an independent observer for both surgeons. There was a statistically significant difference in the side cut corneal healing pattern between the two groups (*P* value < 0.05), and healing is more evident in the femtosecond group (Figure 2).

52% (13 eyes) of femtosecond assisted DALK cases showed wound healing patterns Grades 3 and 4 while only 12% (3 eyes) of manual DALK cases showed the same grades of wound healing (Table 3).

## 4. Discussion

The use of the femtosecond laser in DALK avoids manual trephination and allows more precise identification of tissue depth and insertion of the air needle by following the plane between the lamellar and posterior laser side cuts. Injection of air at this precisely predefined pre-Descemet plane may facilitate the big bubble formation with full baring of DM [9, 10]. Using the FSL to create shaped wound configurations in DALK may combine the mechanical and wound healing advantages found for stepped corneal wounds in PKP with the advantages of the lamellar surgery [11].

As variability in stromal thickness in eyes with advanced keratoconus, ectasia, or dense and deep stromal scars may limit the ability of the femtosecond laser to produce a uniform lamellar plane, we used the FS laser only to create the side cut both in donor and in recipient cornea, while leaving a minimal amount of residual corneal tissue. With this we tried to control the potential risk of creating a large buttonhole or uncontrolled Descemet membrane perforation with the femtosecond laser. In our study, femtosecond laser was programmed to leave a residual stroma according to the pachymetry of each case. Manual dissection of the posterior lamella assisted by air injection (big bubble technique) was chosen, as it allows the surgeon to create a lamellar plane parallel to the more regular posterior corneal surface as opposed to the front surface.

In addition to its advantage in facilitating the DALK procedure, using the FSL to create corneal-shaped wound configurations offers the advantages of better donor-recipient fit with increased surface area contact, which may accelerate wound healing [12, 13].

The mechanical stability of the mushroom configuration (larger anterior diameter cut) created using the FSL has been shown to be superior to traditional straight cuts [13]. In addition, it might have an advantage in keratoconus cases and extensive corneal scars because it provides a larger amount of donor-recipient tissue to interact for the purpose of corneal wound healing consistency [13].

In this study, we compared the outcomes after FSL-assisted mushroom configuration with manual trephine straight edge configuration DALK. Mean postoperative keratometric cylinder, uncorrected distant visual acuity (UCDVA), and best corrected distant visual acuity (BCDVA) were comparable between both groups. FSL and manual trephine DALK techniques provided patients with significantly improved vision postoperatively but the FSL group achieved this improvement faster (mean UCDVA is better at 1 month in the FSL group). The greatest improvement in mean BCDVA occurred at 1 year in both groups. Although it was better in the FSL group at 1 year, BCVA was not significantly different between either group at 1 month, 6 months, and 1 year (*P* = 0.118, *P* = 0.262, and *P* = 0.965, resp.). UCDVA was not significantly different between either group at 1 month, 6 months, and 1 year (*P* = 0.308, *P* = 0.801, and *P* = 0.757, resp.).

Corneal cylinder (topographic) was not significantly different between either group at 1 month, 6 months, and 1 year (*P* = 0.843, *P* = 0.467, and *P* = 0.180, resp.).

The main finding of this investigation was the observation of an evident and statistically significant difference in the side cut corneal healing pattern between the two groups (*P* < 0.05) as observed and graded by the slit lamp appearance by an independent observer, and healing is more evident in the femtosecond group.

The reasons for the femtosecond assisted DALK to show a more active wound healing leading to leucomatous wound could be either due to the larger area of contact between the donor and recipient tissues and/or due to femtosecond laser related biological activation of the corneal tissues, which should be related to the level of energy used for the creation of the side cut; 52% (13 eyes) of femtosecond assisted DALK cases showed wound healing patterns Grades 3 and 4 while only 12% (3 eyes) of manual DALK cases showed the same grades of wound healing.

Although it was previously suggested that using FSL may accelerate suture removal due to faster wound healing related to a better donor-recipient fit with the increased surface area contact [13, 14], in no study performed formerly has it been reported any consistent evidence that such enhanced corneal wound healing actually exists. The results of the present study demonstrate that different and more evident wound healing patterns do exist in Fs-DALK. According to the outcomes of this investigation, earlier suture removal might be possible in FS assisted cases once evidence of initial scarring is observed along the slit lamp biomicroscopic postoperative evaluation.

## 5. Conclusions

This study concluded that Fs-DALK is followed by an increase in the wound healing pattern as observed by clinical biomicroscopy. However, Fs assisted and manual techniques show comparable visual and refractive outcomes at one year of the surgery. The FSL group achieved a significantly improved visual and refractive outcome in terms of UCDVA only at 1 month, an outcome that in our opinion should be considered to be anecdotal. The differences in the wound healing patters in Fs-DALK should be considered to be relevant as the higher levels of the scale used in this investigation imply cosmetic imbalance to the patient, especially in darkly pigmented eyes.

To the best of our knowledge this is the first report in which an increased wound healing response in corneal grafting surgery following femtosecond assisted techniques is demonstrated. Such finding may have implications in the indication of Fs laser in the surgery of keratoconus and in the indication of suture removal following this procedure.

## Figures and Tables

**Table 1:** Analysis of visual outcomes.

Time	Femtosecond DALK	Manual DALK	*P* value
UCDVA (mean)			
1 month	0.17	0.14	0.308
6 months	0.20	0.23	0.801
1 year	0.18	0.20	0.757
BCDVA (mean)			
1 month	0.30	0.39	0.118
6 months	0.45	0.52	0.262
1 year	0.55	0.54	0.965

UCDVA: uncorrected distant visual acuity; BCDVA: best corrected distant visual acuity.

**Table 2:** Analysis of corneal cylinder.

	Femtosecond DALK	Manual DALK	*P* value
1 month	5.16 (1.03–13.58)	5.30 (1.09–10.01)	0.843
6 months	4.60 (1.13–8.47)	4.79 (1.26–20.20)	0.467
1 year	5.43 (1.00–10.27)	4.62 (0.49–13.70)	0.180

**Table 3:** Analysis of side cut corneal wound healing pattern.

			Femtosecond DALK (% of eyes)	Manual DALK (% of eyes)
Grade 0	Transparent scar	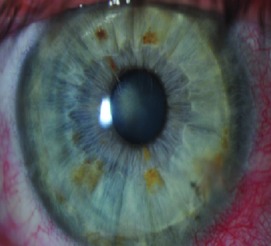	16% (4 eyes)	16% (4 eyes)

Grade 1	Faint healing opacity	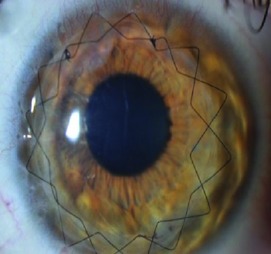	8% (2 eyes)	8% (2 eyes)

Grade 2	Evident healing opacity	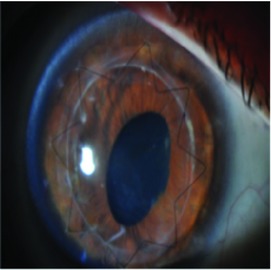	24% (6 eyes)	64% (16 eyes)

Grade 3	Significant opacity with some cosmetic imbalance	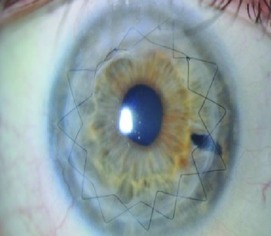	40% (10 eyes)	12% (3 eyes)

Grade 4	Highly significant opacity with very significant cosmetic imbalance	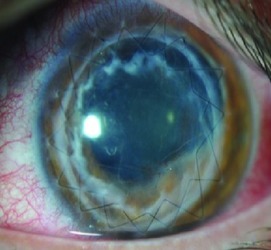	12% (3 eyes)	0%
